# Evidence of Antidepressive Effects of a *Wakan-yaku*, *Hochuekkito*, in Depression Model Mice with Learned-Helplessness Behavior

**DOI:** 10.1155/2013/319073

**Published:** 2013-12-23

**Authors:** Michihisa Tohda, Salin Mingmalairak

**Affiliations:** ^1^Division of Medicinal Pharmacology, Institute of Natural Medicine, University of Toyama, Toyama 930-0194, Japan; ^2^Wakan-yaku Theory-Based Integrated Pharmacology, Graduate School of Innovative Life Science, University of Toyama, Toyama 930-0194, Japan

## Abstract

*Wakan-yaku* is a type of Japanese and Sino traditional, systematized medical care that has been practiced for hundreds of years. This medicinal system includes many antidepressive prescriptions. One of the candidates is *Hochuekkito*, although experimental evidence has not yet been established clearly. To obtain evidence, a depression model of learned-helplessness (LH) mice was used. Based on the score of escape failure, an index of the depression degree, mice with a depressive condition were selected to assess *Hochuekkito*'s effects. This selection was significant and effective in the following two points: evaluation of the drug effect under disease conditions and minimization of the number of animals. Treatment with *Hochuekkito* (1 and 5 g/kg p.o.; estimated galenical amount) for 14 days significantly decreased the depression index, the number of escape failures, and desipramine (10 mg/kg p.o.) suggesting that *Hochuekkito* has an antidepressive effect.

## 1. Introduction

Since the discovery of the antidepressive effect of imipramine in the 1950s, clinical antidepressants have been developed with monoamine transporters as the target molecules [1]. Meanwhile, many “*Wakan-yaku* prescriptions” have been traditionally used to treat depression. By studying the action mechanism of these *Wakan-yaku* prescriptions, elucidation of the generation mechanism of depression and the development of new antidepressants are expected; however, there is little experimental evidence for the antidepressive effects of *Wakan-yaku*.

The learned-helplessness (LH) behavior of experimental animals caused by inescapable footshocks is one behavioral phenotype relevant to depression [1, 2]. Since this behavior can be ameliorated by antidepressants, LH animals have been used for studying the pathophysiology of depression and the actions of antidepressants [3, 4]. LH model animals can evaluate a drug effect under more pathological conditions than the forced swimming test and tail suspension test, which are evaluated by correlation. It has been also reported that the forced swimming itself is a strong and sustained stress [5]. Therefore, in this study, the effect of a *Wakan-yaku* prescription, *Hochuekkito*, was evaluated using LH mice.

*Hochuekkito* has antidepressant-like effects in the forced swimming test [6]. A description of *Hochuekkito* (*Bu Zhong Yi Qi Tang* in Chinese) first appeared in Neiwaishang Bianhuolun, which was written by Dong Yuan Li in 1247. At that time, people living around Li had lost their physical strength because of malnutrition during war; therefore, Li developed *Hochuekkito* for the purpose of “improvement of the digestive system (“*hochu*” in Japanese) and promoting vigor (“*ekki*”)” [7]. Then *Hochuekkito* was sometimes used for the treatment of depressive symptoms with a lack of appetite in *Kanpo*-traditional medicines. Antidepressants cause a lack of appetite as a side effect. *Hochuekkito* seems to be desirable to treat depression with a lack of appetite; however, there is little evidence for the antidepressant action of *Hochuekkito*, with the result that it is rarely used clinically; therefore, we examined the antidepressant effect of *Hochuekkito* using the LH model.

## 2. Materials and Methods 

### 2.1. Hochuekkito Preparation

*Hochuekkito* was prepared by mixing ten dried crude forms of herbal components (purchased from Tochimoto Tenkuido Co., Ltd., Osaka, Japan) and preparing an extract from the mixture as previously reported [8]. The qualitative certification was done by 3D-HPLC (see Supplementary Figure 1 available on line at http://dx.doi.org/10.1155/2013/319073) under the same condition in previous report [8]. In brief, 49 g of a ten galenical mixture was added to 500 mL distilled water and extracted at 100°C for 60 min. Galenicals were removed while the solution was still hot. The extracted solution was filtered and freeze-dried to obtain dry extract powder. Part of the frozen extract was lyophilized to assess the yield (34%). The dose of *Hochuekkito* was shown by the estimated galenical amount using this yield value. The quality of this drug is controlled by measuring the contents by high-performance liquid chromatography (HPLC) (data not shown). Before the HET extract was applied to cultured cells, it was centrifuged to remove insoluble matter and filter-sterilized.

### 2.2. Animals

Ten-week-old male ICR mice (Japan SLC Inc., Shizuoka, Japan) weighing 38–40 g were housed in groups of eight per cage under standard conditions: 25 ± 1°C ambient temperature; 65 ± 5% relative humidity; 12 h light/dark cycle (lights on from 07:30 to 19:30). They had *ad libitum* access to food and water and were allowed to acclimatize to these housing conditions for 1 week prior to the experiments. Care and use of the animals was in accordance with the Guiding Principles for the Care and Use of Animals in the Field of Physiological Science of the Physiological Society of Japan and the Institutional Animal Use and Care Committee of University of Toyama. Studies were designed to minimize pain and the number of animals used.

### 2.3. Learned-Helplessness Paradigm

The paradigm and experimental conditions used to induce LH behavior in mice are the same as previously reported [9]. In the avoidance test conducted on day 4, mice were selected for the experiments as LH animals if escape failed more than 10 times.

### 2.4. Drug Administration

All drugs were dissolved and diluted with water to the appropriate concentration. *Hochuekkito* at doses of 1 or 5 g/kg and desipramine (Sigma-Aldrich, St. Louis, MO, USA) at a dose of 10 mg/kg were administered orally through a feeding needle inserted down the throat of the mice in a volume of 10 mL/kg body weight. Drug was administered once daily for 14 days, starting on day 5. On day 18, the mice were tested for escape ability under escapable shock conditions 60 min after the last administration of drug.

### 2.5. Statistical Analysis

All data are presented as mean ± S.E.M. Two-way repeated-measure analysis of variance (ANOVA), followed by the post hoc analysis test, was used for the statistic evaluation of behavioral data obtained from the avoidance test.

## 3. Results 

**Figure 1:**
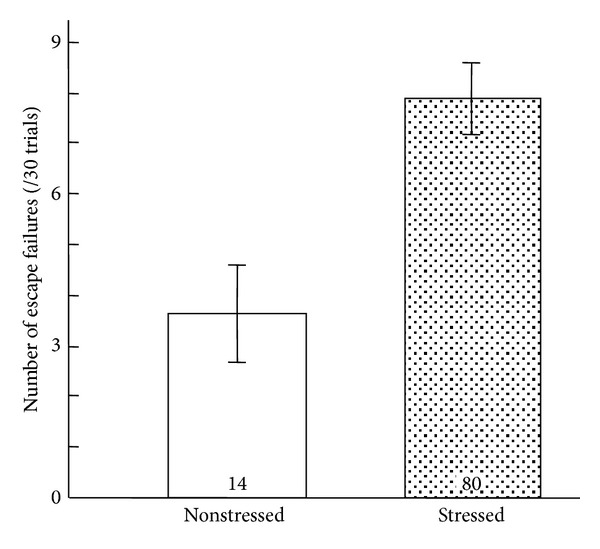
Learned-helplessness paradigm to develop depression model mice. The number of escape failures of the footshock-naïve control (nonstressed) and animals that had received inescapable footshock (stressed) was analyzed. The stressed animals received footshock under inescapable conditions once a day for 3 consecutive days. The avoidance test was performed on days 4 and 18 under escapable conditions. The avoidance test on day 18 was conducted 60 min after the last drug administration. The data are expressed as the mean ± S.E.M.

**Figure 2:**
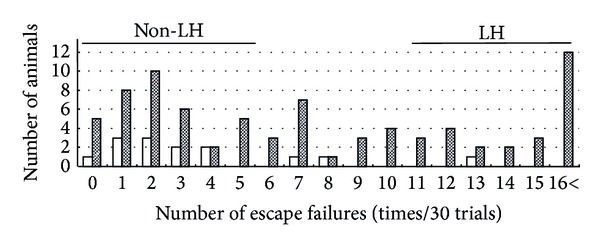
Phenotypic difference of mice in LH behavior test. The number of escape failures by the footshock-naïve control (open bars) and animals that had received inescapable footshock (closed bars) was analyzed. This diagram shows the distribution of the number of failures in this avoidance test. Animals that had received inescapable footshocks in the training session were classified into two groups: the LH group with failure scores no less than 10 as depressive mice and the non-LH group with failure scores no more than 5 as stress-resistant mice.

**Figure 3:**
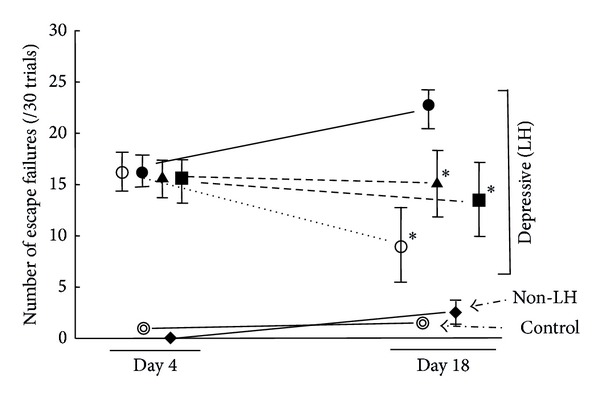
Effects of *Hochuekkito* and desipramine in the LH behavior test using depressive conditioned mice, non-LH mice and naïve control mice. The animals, except for naïve control (⊚), received inescapable footshock once a day for 3 consecutive days. The avoidance test was performed on day 4 and 18 under escapable conditions. One g/kg/day *Hochuekkito* (▲), 5 g/kg/day *Hochuekkito* (■), 10 mg/kg/day desipramine (◯) or water as a vehicle (*⚫*) was* p.o.* administered in depressive conditioned mice once daily for 14 days from day 5. Water was *p.o.* administered also in non-LH condition (◆) and in naïve control (⊚). The avoidance test on day 18 was conducted 60 min after the last drug administration. The data are expressed as the mean ± S.E.M. (*n* = 5  per group). Significance: *P < 0.05  versus vehicle-treated mice on day 18 (*⚫*) (two-way repeated ANOVA followed by the Student-Newman-Keuls test).

As shown in Figure 1, the failure scores of the animals exposed to inescapable footshock stress (7.875 ± 0.714, *n* = 80) were higher than those of naïve control animals (3.642 ± 0.941, *n* = 14); however, the difference between these values was not enough to easily evaluate the effect of drugs. Many animals seem to be required for evaluation under this experimental condition. In addition, the results of LH animals did not seem to show normal distribution: normal animals and those with depression seemed to coexist (Figure 2). Therefore, the animals were divided into LH mice (depression model) and non-LH (depression-resistant model) based on the escape failure scores at the end of the avoidance test on day 4 (Figure 2). Out of the animals that had been exposed to inescapable footshock on day 1, around 30% of the animals exhibited LH behavior in the avoidance test conducted on day 4 (Figure 2). This result was consistent with previous reports [10, 11]. The LH animals were then randomly divided into 4 groups. Each group received oral administration of 1 or 5 g/kg *Hochuekkito*, 10 mg/kg desipramine, or water as vehicle once daily for 14 days from day 5. As shown in Figure 3, when vehicle-treated LH animals were subjected to footshock under an escapable condition on day 18, they exhibited a deficit in escape performance. On the other hand, desipramine (10 mg/kg/day), a typical antidepressant, significantly decreased the number of escape failures in post hoc analysis (*P* = 0.002). This significant difference was obtained by the data from only five animals each group. These results suggest that this method to evaluate the antidepressant effect using depressed animals has high sensitivity and can assess the effect with the minimum number of animals. Effects of *Hochuekkito* (1 and 5 g/kg/day) also showed a significant difference versus vehicle-treated LH animals in post hoc analysis (1 g/kg/day: *P* = 0.014, 5 g/kg/day: *P* = 0.027), although the number of escape failures did not improve (Figure 3).

## 4. Discussion

This report suggests the importance of using depression model animals to evaluate drugs for depression. These model animals, represented by LH mice in this study, could be divided into animals with and without depression using this behavioral pharmacological method. The number of animals with depression could be increased by applying higher strength electrical stimulation, but this is unfavorable from the viewpoint of animal welfare. Around 30% of animals showed depression symptoms under the conditions of this experiment. Only animals in a depressed condition were given drugs to evaluate the antidepressant effect.

The “general pharmacological order” to evaluate the effects of drugs and/or treatments using all of the animals in the group is inefficient. In the general pharmacological order manner of this LH method, the difference in escape failure score between stressed and nonstressed mice was small (7.9 versus 3.6) (Figure 1), and it was doubtful whether the score showed normal distribution (Figure 2). Many animals are necessary for drug evaluation. Since medicines are used for disease treatment, it is logical to use only for experiments animals that have the disorder being evaluated by experimental diagnostics. Indeed in this experiment, drug evaluation was possible using the minimum number of animals efficiently by choosing depression conditioned animals; five in each group was sufficient. Desipramine, a typical antidepressant, improved the index of depression. We previously reported the similar effect of imipramine [9], whereas the index became worse in water-administered animals with depression. These results suggest that this strategy is highly useful to evaluate the drug effect using the minimal number of animals.

Based on the above-mentioned points, the effect of *Hochuekkito* was evaluated as an antidepressant. The results showed the antidepressive ability of *Hochuekkito* since the index score of depression was significantly lower than that in the water-administrated group, although the score was not improved by 14 days of treatment, as shown in desipramine. Therefore, this result may provide evidence that *Hochuekkito* is an antidepressant or at least prevents pathologic aggravation. A previous report that showed evidence of *Hochuekkito's* antidepressive potential used the forced swimming test [6]; however, it has been reported that the forced swimming itself is a strong and sustained stress [5]. In addition, the forced swimming test is based on general pharmacological order. It does not use animals with depression, and it is merely a convincing rating system based on correlation. This experimental method reflecting depression is useful and valuable to obtain pharmacological evidence to support *Wakan-yaku* prescriptions, not only *Hochuekkito*, and to elucidate the action mechanism and active ingredients.

It is important to use the depressed state animal for evaluation of an antidepressant effect. In addition to the LH model, a chronic mild stress (CMS) model animal is also used for the evaluation [12]. The selection of the depressive state animals, in also the CMS model, may allow us to get significant information under the decreased number of animals. It is a further important point of view for animal protection. In alternative medicine, there are many materials from which an antidepressant effect is expected. For example, the ingredients of *royal ielly* [13] and *Perillae Herba* [14] have been reported. In addition, another *Wakan-yaku* prescription Juzentaihoto (*Shi Quan Da Bu Tang *in Chinese) is also used to expect the clinically antidepressant effect in *Kanpo*-traditional medicines. Although there are many reports about Juzentaihoto for cancer treatment and immune functions [15], the scientific evidence about the antidepressant effect is not provided yet. Both Juzentaihoto and *Hochuekkito* are constructed by ten kinds of crude drugs, and five kinds are the same among the ten. The roles of the common 5 of crude drugs group and the “personality” induced by the peculiar 5 of crude drugs group in the efficacies of *Hochuekkito* and Juzentaihoto will be also interesting. For the detailed studies such as the difference in mechanism, the examination based on this way will be an advantage. Authors have preliminary results for  *Hochuekkito* about the stimulation to alimentary system by the peculiar 5 of crude drugs group having the prescription-specific contribution of *Hochuekkito* for an antidepressive effect and the inhibitory effect of prior administration of an antidepressant such as imipramine because of the downregulation of alimentary system. The detailed mechanisms of that will be a further report by using this way of experiment and the MRI diagnostics.

## Supplementary Material

The 3D-HPLC pattern of Hochuekkito. The Hochuekkito extract solution was filtrated with a membrane filter (0.22 mm) and then submitted for HPLC analysis. We usually take and keep the 3D-HPLC fingerprints at every experiment, although the fixed-quantity about the particularly specific ingredient does not reach. We only confirm that it does not have the big difference on the fingerprints in this time. In the future experiment, if we get some unexpected results, these “3D-HPLC fingerprints with detail electric data in behind” will give us novel welcome knowledge by analysis of these. The following peaks are described in this figure: (1) glycyrrhizic acid (Glycyrrhiza Radix), (2) saikosaponin b2 (Bupleuri Radix), (3) isoliquiritin (Glycyrrhiza Radix), (4) apioisoliquiritin (Glycyrrhiza Radix), (5) hesperidin (Citri Leiocarpae Exocarpium), (6) narirutin (Citri Leiocarpae Exocarpium), (7) 3-(3-hyroxy-4-methoxyphenyl)-2-(E)-prepenoic acid? (Cimicifugae Rhizoma) with peaks of liquiritin and apioliquiritin (Glycyrrhiza Radix) in both side. Other isolated elements will also detectable in this figure and in sensitivity enhanced chart (data not shown), although these are not annotated.
