# Supplementary material for: Evidence of Antidepressive Effects of a Wakan-yaku, Hochuekkito, in Depression Model Mice with Learned-Helplessness Behavior
Source: Evid Based Complement Alternat Med. 2013 Dec 23;2013:319073. doi: 10.1155/2013/319073 (PMC3884744; doi:10.1155/2013/319073)
Supplement: Supplementary file 1 — The 3D-HPLC pattern of Hochuekkito. The Hochuekkito extract solution was filtrated with a membrane filter (0.22 mm) and then submitted for HPLC analysis. We usually take and keep the 3D-HPLC fingerprints at every experiment, although the fixed-quantity about the particularly specific ingredient does not reach. We only confirm that it does not have the big difference on the fingerprints in this time. In the future experiment, if we get some unexpected results, these “3D-HPLC fingerprints with detail electric data in behind” will give us novel welcome knowledge by analysis of these. The following peaks are described in this figure: (1) glycyrrhizic acid (Glycyrrhiza Radix), (2) saikosaponin b2 (Bupleuri Radix), (3) isoliquiritin (Glycyrrhiza Radix), (4) apioisoliquiritin (Glycyrrhiza Radix), (5) hesperidin (Citri Leiocarpae Exocarpium), (6) narirutin (Citri Leiocarpae Exocarpium), (7) 3-(3-hyroxy-4-methoxyphenyl)-2-(E)-prepenoic acid? (Cimicifugae Rhizoma) with peaks of liquiritin and apioliquiritin (Glycyrrhiza Radix) in both side. Other isolated elements will also detectable in this figure and in sensitivity enhanced chart (data not shown), although these are not annotated. [file 319073.f1.pdf]

Supple. Fig. 1 (Tohda and Mingmalairak)

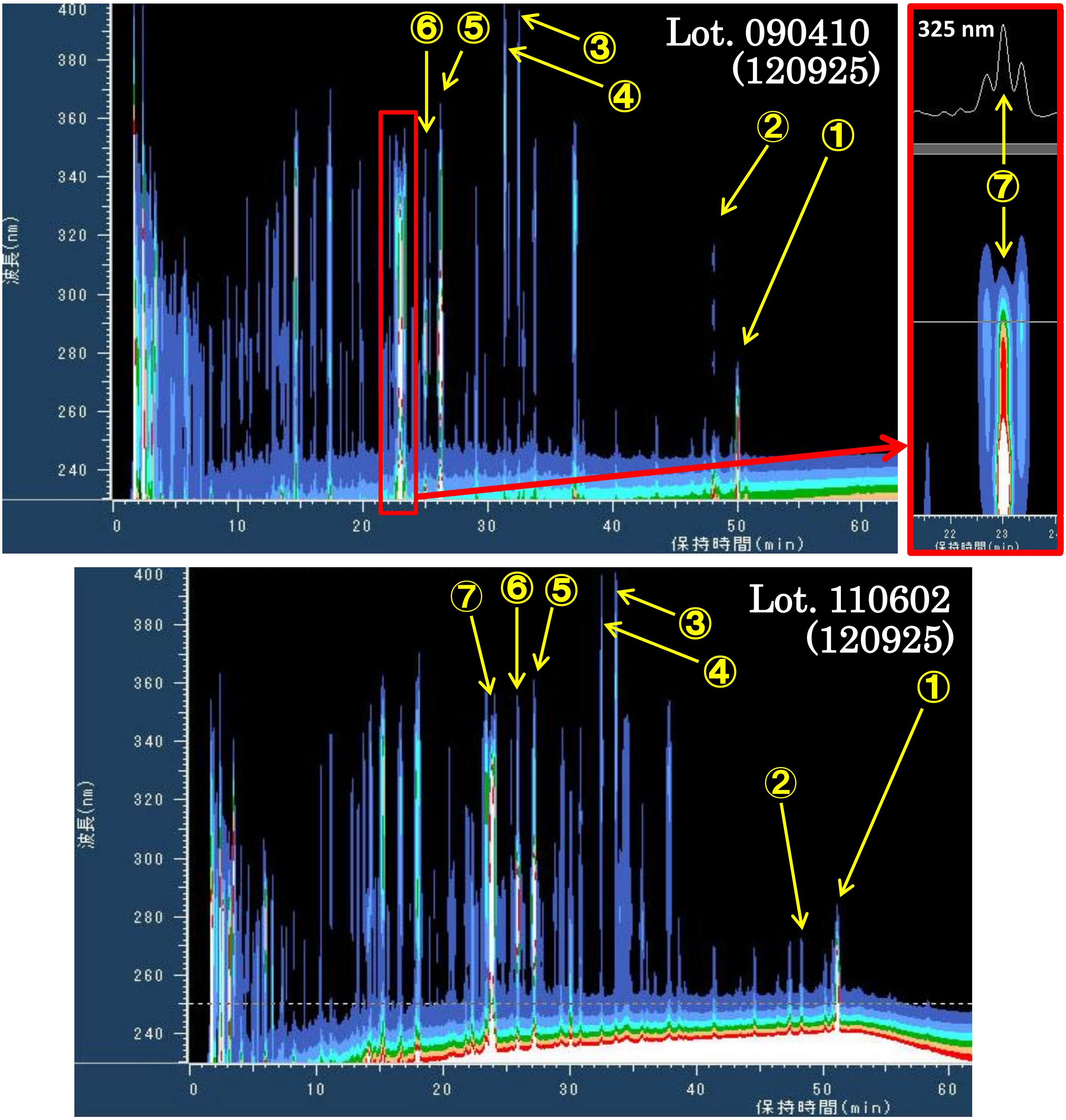

Suppl. Fig. 1. The 3D-HPLC pattern of *Hochuekkito*. The *Hochuekkito* extract solution was filtrated with a membrane filter (0.22 mm) and then submitted for HPLC analysis. We usually take and keep the 3D-HPLC fingerprints at every experiment, although the fixed-quantity about the particularly specific ingredient does not reach. We only confirm that it does not have the big difference on the fingerprints in this time. In the future experiment, if we get some unexpected results, these "3D-HPLC fingerprints with detail electric data in behind" will give us novel welcome knowledge by analysis of these. The following peaks are described in this figure: (1) glycyrrhizic acid (Glycyrrhiza Radix), (2) saikosaponin b2 (Bupleuri Radix), (3) isoliquiritin (Glycyrrhiza Radix), (4) apioisoliquiritin (Glycyrrhiza Radix), (5) hesperidin (Citri Leiocarpae Exocarpium), (6) narirutin (Citri Leiocarpae Exocarpium), (7) 3-(3-hyroxy-4-methoxyphenyl)-2-(E)-prepenoic acid? (Cimicifugae Rhizoma) with peaks of liquiritin and apioliquiritin (Glycyrrhiza Radix) in both side. Other isolated elements will also detectable in this figure and in sensitivity enhanced chart (data not shown), although these are not annotated.
